# Facemask and social distancing, pillars of opening up economies

**DOI:** 10.1371/journal.pone.0249677

**Published:** 2021-04-20

**Authors:** Ali Najmi, Sahar Nazari, Farshid Safarighouzhdi, C. Raina MacIntyre, Eric J. Miller, Taha H. Rashidi

**Affiliations:** 1 Research Centre for Integrated Transport Innovation, School of Civil and Environmental Engineering, The University of New South Wales, Sydney, Australia; 2 School of Engineering, Macquarie University, Sydney, Australia; 3 School of Chemical Engineering, University of New South Wales, Sydney, NSW, Australia; 4 Arizona State University College of Health Solutions, Phoenix, Arizona, United States of America; 5 Kirby Institute, Faculty of Medicine, The University of New South Wales, Sydney, New South Wales, Australia; 6 Department of Civil & Mineral Engineering, University of Toronto, Toronto, ON, Canada; Universidad Nacional de Mar del Plata, ARGENTINA

## Abstract

The COVID-19 pandemic has caused severe health and economic impacts globally. Strategies to safely reopen economies, travel and trade are a high priority. Until a reliable vaccine is available, non-pharmaceutical techniques are the only available means of disease control. In this paper, we aim to evaluate the extent to which social distancing (SD) and facemask (FM) use can mitigate the transmission of COVID-19 when restrictions are lifted. We used a microsimulation activity-based model for Sydney Greater Metropolitan Area, to evaluate the power of SD and FM in controlling the pandemic under numerous scenarios. The hypothetical scenarios are designed to picture feasible futures under different assumptions. Assuming that the isolation of infected cases and the quarantining of close contacts are in place, different numerical tests are conducted and a full factorial two-way MANOVA test is used to evaluate the effectiveness of the FM and SD control strategies. The main and interactive effects of the containment strategies are evaluated by the total number of infections, percentage of infections reduction, the time it takes to get the pandemic under control, and the intensity of active cases.

## 1. Introduction

Enhanced surveillance and testing, case isolation, contact tracing and quarantine, social distancing (SD), teleworking, travel bans, closing businesses, school closure, and lockdown are the most common strategies implemented worldwide for slowing down COVID-19 spread. While combinations of these control strategies are currently in place in many countries, continuation of indefinite lockdown is not feasible economically, and long-term restrictions may result in mental and psychological distress [1]. Many authorities are thus looking at options for easing or lifting of the restrictions. SD and facemasks (FM) are potential control strategies that may enable lifting the restrictions.

SD is highly emphasized as a pre-requisite of easing restrictions on many businesses and social activities as it effectively reduces the infection rate [2]. However, compliance with SD is not usually perfect and is affected by poor compliance with guidelines, SD fatigue over time, and urban high-density zones (such as public transport) where SD compliance is unfeasible. COVID-19 guidelines issued by WHO and other agencies recommend SD of 1–2 m but vary on use of FM [3]. This reflects uncertain evidence about the transmission mode of severe acute respiratory syndrome coronavirus 2 (SARS-CoV-2). A recent systematic review and meta-analysis of masks against beta-coronaviruses found FM reduce infection by 67% effective (aOR 0·33, 95% CI 0·17–0·61) [4]. The study supports universal FM, which can reduce the rate of infection (flatten the curve), even with modestly effective masks [5]. The studies which have modelled SD and FM use have used deterministic mathematical models which cannot account for the behaviour of individuals in different settings such as within households or in public [5]. The studies have been observational epidemiologic studies with a focus on individual and micro-level settings. Translating this evidence to population health requires modelling. The potential capacity of FM and SD to mitigate the pandemic at a population-level, where complications of the system, network, infrastructure, and heterogeneity in the population are accounted for, has been overlooked. Accounting for these complications in the model allows modelers to provide a realistic view of the system. Activity-based models (ABM) can account for these.

To model COVID-19 spread and evaluate the population-level performance of the SD and FM, we used the activity-based SydneyGMA model system. SydneyGMA is an established ABM for the city of Sydney, Australia [6]. It provides a suitable platform to assess the “relative” impacts of the various SM and FM policies. SydneyGMA can capture the dynamics of disease spread combined with the heterogeneous mixing and social networks of individuals (people). SydneyGMA also models the interactions of individuals within a household and therefore the interconnection between travel decisions and activities of different household members.

The results and findings of this paper are of significance and innovation in three ways. First, we provide guidance to public health agencies worldwide as they consider slowing down disease spread and easing of restrictions. Note that the aim of this paper is not to find a strategy to afford complete protection from infection; instead, it aims to suggest sufficient intervention levels of SD and FM that maintain different system-level evaluation measures at acceptable levels. Second, the system-level performance of SD and FM use of this study is different than SD and FM efficiencies that have been reported in the literature. The literature is limited to observational epidemiologic studies on individual and micro-level settings with particular limitations including working on very small population size, considering solely the confirmed cases and their family members, healthcare settings, trial period of usually two weeks etc. This might be logical in the laboratory settings, though given the challenges associated with measuring the efficiency at the whole lifecycle of pandemic, many influential parameters in reality, and over the whole population is neglected. Evaluation of FM and SD performance at the population-level using a system which resembles the society in reality is the focus of this paper. Third, we present marginal benefits of the SD and FM control strategies, not the absolute magnitude of the benefits using our model which reflects, like all models, in part the ground truth. The microsimulation ABM, like all microsimulation platforms, once calibrated to an acceptable level, is useful when relative benefits of hypothetical scenarios and policies are studied. Given the extensive complication associated with the real world and the expectation from the microsimulation platform to reflect the infrastructure, network configurations and population heterogeneity, one small change in the system can result in significant variations in the results. This is the reason supporting the argument that ABMs are only useful to assess the relative consequences of decisions once they are run many times to obtain robust and stable results. S1 File provides a descriptive example on the sensitivity of a simulation model to the parameters considered. The point of this example is to clarify the significance of looking at the relative gap between scenarios, conditional on the assumption that the model reflects the behaviour of the system to an acceptable level. This is a common practice and understating of all microsimulation planning platforms.

### 1.1. Aim

To identify effective intervention levels of SD and FM for pandemic control.

## 2. Methodology

This section briefly explains the ABM that is used for scenario simulations. Then, the control strategies are discussed followed by introducing different measures to evaluate the control strategies. After that, the statistical approach for conducting the analysis will be elaborated.

### 2.1. SydneyGMA model

The activity-based disease transmission model in this paper is built on an ABM developed for the Sydney GMA, called SydneyGMA model, which has several properties that are valuable for analysing the effectiveness of COVID-19 control strategies. Firstly, SydneyGMA uses the Travel/Activity Scheduler for Household Agents (TASHA), an operational, state-of-the-art model of daily travel and out-of-home activity participation that considers both individual activities as well as joint household activities, along with a full range of within-household interactions [7–12]. In addition to Sydney, TASHA has been applied in Toronto, Canada, where it is the operational model for Toronto transportation planning agencies [13], Finland [14], and Temuco, Chile [15]. Secondly, mode choice is computed for each household individually, and interactions among household members using their vehicle on individual or joint trips are captured, as well their usage of other modes of travel, notably transit. Thirdly, the model “assigns” transit (PT) trips to explicit paths through the transit network, enabling different components of transit trips (including in-vehicle, walking to/from transit, and waiting and transferring) to be estimated and considered as potential locations for disease spread. Therefore, utilising the SydneyGMA augments disease spread modelling by accounting for potential locations of disease spread and more accurately modelling interactions among household members as a result of adjustments to their daily activities. Building on SydneyGMA, an activity-based disease transmission model is developed (see [16]) which simulates the disease spread in the system; further details of the ABM and the disease transmission model are provided in S2 File. The disease transmission model has been calibrated against daily and cumulative infected cases observed in the Sydney Metropolitan area [17]. Note that the capability of the model in reproducing the observed statistics as well as its sensitivity to various control strategies have been illustrated in Najmi et al. [6]. Using this model, different numerical tests are conducted using SydneyGMA, allowing the transmission of the disease among individuals. In each of the tests, the performance of imposing different levels of SD and FM control strategies is investigated. The parameters transmission parameters for the ABM are provided in Table 1.

**Table 1:** Calibrated COVID-19 transmission parameters in the SydneyGMA.

Parameters	value
Infection probability out-of-home (per contact)	0.041
Infection probability in-home (per day per infected case)	0.055
Case isolation probability (per day)	0.12
Base contact number (per activity)	2
Incubation period	5
Latent period	3
Contact number in public transport vehicle	13.85
Starting number of infections	4
Correction factor of infection probability of individuals with manufacturing, professional and other occupations compared to general and sale occupations	1.26
Effectiveness of facemask in reducing transmission out-of-home	0.67
Effectiveness of facemask in reducing transmission in-home	0.79

### 2.2. Control strategies

Knowing that SD and FM can reduce the rate of spread, in scenarios other than a total lockdown, expecting all to use FM and maintain the recommended SD may be too optimistic. Therefore, the benefits of partial compliance of people under control strategies to support opening of businesses are yet to be measured. We also evaluated the role of FM and SD and their interactions in controlling disease spread. We assume that case isolation (CI) and quarantine of traced family contacts of infected cases are in place across all experiments. People are isolated if they become infected and traced which is followed by quarantining their close contacts. We assumed that the infected cases get isolated based on the daily case isolated probability given in Table 1. Also, we assumed that all the family members of the isolated cases are quarantined for 14 days. As our intention is to evaluate effectiveness of the control strategies in opening up businesses and other activities, we assume that everyone conducts their daily activities as it was pre-COVID-19 unless they are isolated or quarantined. In other words, in the model, all businesses, schools, universities, recreational centres etc. are open and there is no lockdown. We do assume that international travel restrictions are in place. Scenario assumptions for each of the control strategies are briefly described below.

#### 2.2.1. Social Distancing (SD)

SD is a key parameter in disease transmission models and affects the rate at which infected people infect susceptible people. We impose SD in our model by reducing all non-household contacts (referred to as compliance level) while the intra-household contacts are kept unchanged in line with the pandemic studies of Chang et al. [18] and Ferguson et al. [19]. SD compliance levels may vary from zero-SD–no compliance- to full lockdown-full compliance. When there is no restriction on movement, the chances of high SD compliance are lower. In this paper, we consider four SD compliance levels of 0%, 30%, 50%, and 70%. For households with traced contacts and isolated cases, we assume that the household members comply with intra-household SD better and assume a 50% reduction in the intra-household contact rate. In households without infected members, there is no reduction in the intra-household contact rate. We assumed the effectiveness of SD in reducing transmission to be applied by reducing the contact numbers. Thus, contact numbers are reduced by increasing the SD level.

#### 2.2.2. Travel Load (TL)

Travel load reflects the level of social mobility of the individuals within Sydney Greater Metropolitan area. We assume that the intra-urban TL is at the same level as before COVID-19 (almost 100%) when all the individuals participate in their activities without restrictions.

#### 2.2.3. Use of Facemasks Out-of-Home (FMOH)

We assumed FMOH levels may vary between 0% to 100%. In this paper we evaluate the disease spread at six FMOH levels of 0%, 20%, 40%, 60%, 80% and 100%.

#### 2.2.4. Use of Facemask in-Home (FMH)

FMH can reduce secondary cases in households with an infected person [20]. We define six FMH levels similar to those for FMOH.

### 2.3. Evaluation measures

The following measures are used to compare the effects of different control strategies, at their various compliance levels:

#### 2.3.1. Total Infections (TI)

The total number of infections in Sydney Greater Metropolitan Area.

#### 2.3.2. Percentage of Reduction (PoR)

The percentage of reduction in the number of cases which is obtained by comparing the number of infections obtained at different SD and FM levels with a scenario where no SD and FM control strategies (both the SD and FM levels are at 0%) are in place. For better understanding of the meaning of one percent reduction in the number of infections, it is worth mentioning that the scenario with no SD and FM strategies generate about 3.75 million infections in the SGMA. Thus, one percent reduction corresponds to about 37.5 thousand infections.

#### 2.3.3. Virus Elimination (VE)

The time it takes, in days, until there is no community transmission where nobody gets infected by the virus unless they have been overseas recently or been in recent contact with other confirmed cases.

#### 2.3.4. Active Case Intensity (ACI)

This is an indicator to evaluate the pressure on the health system and comprises three measures of *Low* (ACI-L), *Medium* (ACI-M), and *High* (ACI-H). ACI-L refers to the number of days that the system experience less than 500 concurrent active cases. Similarly, ACI-M and ACI-H, respectively, refer to the number of days with between 500 and 12,000, and more than 12,000 concurrent active cases. The higher the number of days for ACI-H is, the higher pressure the health system experiences.

#### 2.3.5. In-Home Infection (IHI)

Contrary to the other evaluation measures that are population-level, IHI is household-level and measures the impact of FM use on the rate of infections in the household. The impact of FM use on IHI is already discussed in the literature via observational epidemiologic studies with limited samples [20–22]; however, it has not been explored using a large-scale microsimulation model yet.

### 2.4. Starting points

The starting point at which the FM and SD are imposed, including the daily number of infections and cumulative infections, may influence the performance of the control strategies. To address this issue and to better evaluate the performance of the control strategies in controlling the disease transmission, we impose the FM and SD at different starting conditions brought in Table 2. In line with this, we allow the individuals in the system to progressively get infected without enforcing the SD and FM control strategies until reaching to the starting points; then, the enforcement of the SD and FM control strategies starts. For example, at the starting point 2 at which we apply the SD and FM control strategies, the daily and cumulative infections reach 108 and 699 cases.

**Table 2:** Starting points of different random streams for facemask analysis.

Initial conditions	Daily infections	Cumulative infections
Starting point 1	11	73
Starting point 2	108	699
Starting point 3	1,040	7,975

This research does not seek the absolute magnitude of the benefits at various settings of control strategies; instead, it seeks the estimated marginal means of the strategies and their difference. Therefore, the starting points do not have significant effects on the results. The estimated marginal means explains the mean response for each control strategy, adjusted for any other variables in the model. Also, note that only the infections and the period after the starting points are considered while obtaining the evaluation measures.

### 2.5. Statistical analysis

Considering the number of levels for each of the control strategies and the three starting points, we conducted full factorial experiments. Therefore, a total of 432 singular experiments with different settings were run. In addition, each of the experiments was run three times to address the randomness in the ABM. The averages of the evaluation measures were used for further analysis.

This study utilises two-way multivariate analysis of variance (MANOVA) to test the effects of different SD and FM levels on the virus transmission and some population-level attributes. Two-way MANOVA is often considered an extension of two-way ANOVA suitable for situations where two or more groups are compared across several variables [23]. The MANOVA was used to test the assumption that the control strategy levels significantly influence various population-level evaluation measures. In the MANOVA test, each of the control strategies is regarded as an independent variable while the evaluation measures are dependent variables. To evaluate the appropriateness of the multivariate test of variance the two multivariate test statistics presented in Table 3, namely Pillai’s Trace and Wilks’ Lambda, were conducted to test the statistical significance of the independent variables’ effects on the dependent variables. All the test statistics are statistically significant and, hence, we can confidently reject the null hypothesis of the means of all levels being equal. As a result, the multivariate test of variance is appropriate for use in our study.

**Table 3:** Results of tests to evaluate the appropriateness of the multivariate test of variance.

	Pillai’s Trace			Wilks’ Lambda		
Effect	Value	F	Sig.	Value	F	Sig.
SD	1.897	40.404	0.000	.000	717.993	0.000
FMOH	2.265	19.741	0.000	.000	342.557	0.000
FMH	0.943	20.818	0.000	.030	142.114	0.000

Assessing the effect of each control strategy on the evaluation measures collectively when ignoring the value of the other control strategies is of utmost importance which can be analysed via post-hoc tests; this is conducted in S3 File. In addition to the MANOVA analysis, we plotted several interactive effects of the control strategies and investigates the behaviour of each control strategy at the presence of other ones.

## 3. Results

**Fig 1:**
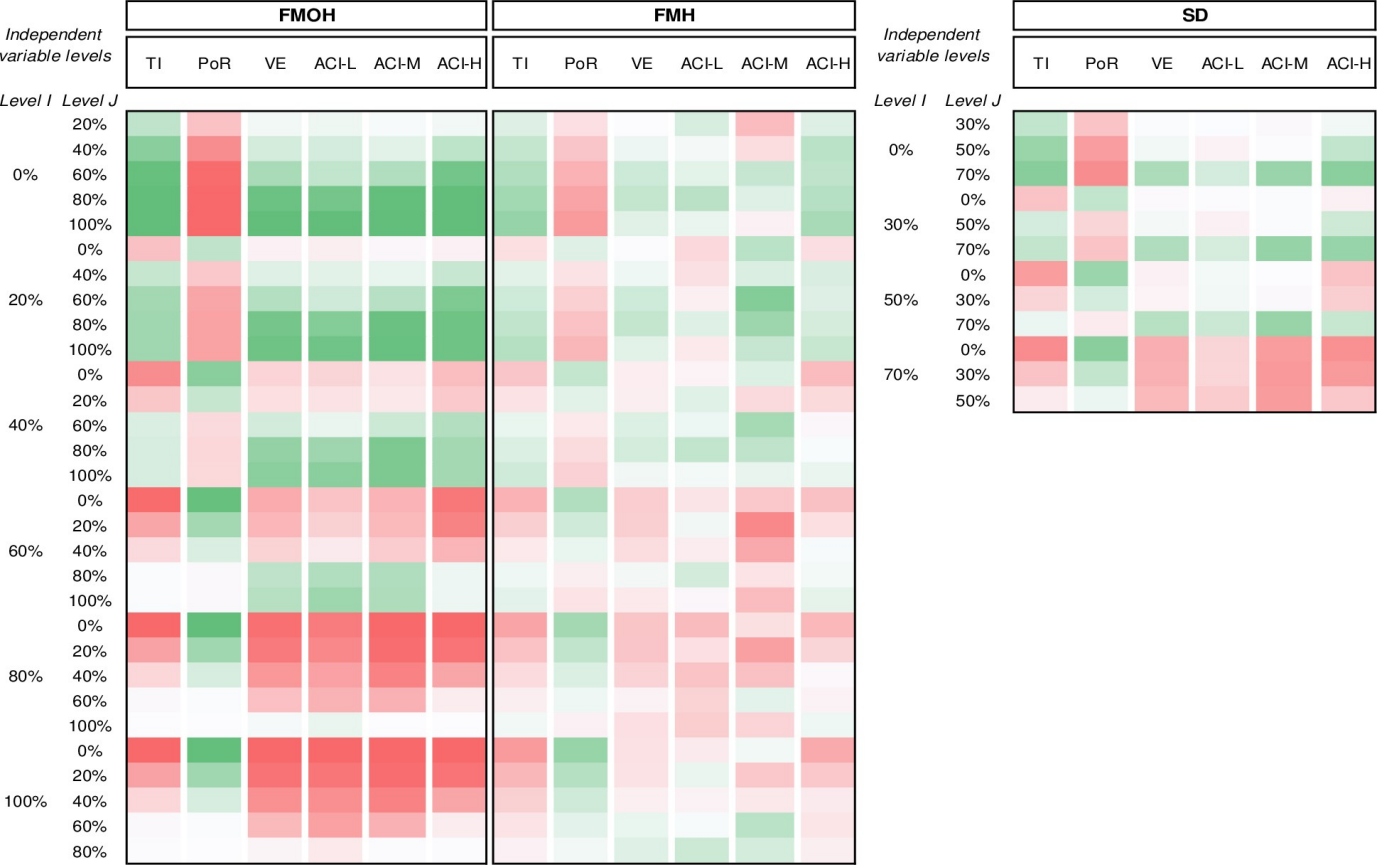
The mean difference (I-J) between different control strategy levels obtained by two-way MANOVA dark green is the maximum and dark red implies minimum; Percentage of Reduction (POR); Total Infections (TI); Virus Elimination (VE); Active Cases Intensity–Low (ACI-L); Active Cases Intensity–Medium (ACI-M); Active Cases Intensity–High (ACI-H).

S3 File presents a pairwise comparison of the different levels of control strategies obtained by post-hoc tests; in each row, two levels, called I and J, are compared regarding their impact on the evaluation measures, as the dependent variables. The content of the pairwise comparisons is transformed to the heatmap in Fig 1 for convenience.

Both the SD and FM control strategies have positive correlations with the TI and PoR; higher SD compliance and FM levels result in greater reduction in the number of infections. FM can reduce infection, in population-level, by 54.4%; of which 32.6% and 21.6%, respectively, belong to the FMOH and FMH. With a compliance level of 70%, SD can reduce infections by 24.7%. Comparing the reduction of infection at all the SD and FMOH compliance levels shows that FMOH use by 40% of people has the same effect as the SD compliance of 70%. Furthermore, the FMOH consistently improves the other performance measures up to the level 80% with no noticeable improvement afterwards.

Another noticeable difference between the SD and FMOH is the VE measure. VE shows a positive and strong relationship with FM. At the compliance levels above 40%, FMOH significantly shorten the average number of days it takes the epidemic to come under control. The FMOH of 40%, 60%, 80% and 100% can shorten the VE period by 119.9, 244.7, 426.4, and 448.9 days, respectively; this shows the exceptional influence of wearing mask by at least 80% of people on shortening the pandemic lifecycle. In contrast, SD does not have statistically significant effects on VE except at its 70% level. SD of 70%, however, can shorten the VE period by 233.9 days which is much less that the corresponding value for the FMOHs of 80%. The FMH influence on VE is also strong but not as much as FMOH. FMH can shorten the VE period by at most 167.2 which is comparable with the FMOH of 50%, if we interpolate the results in S3 File.

The control strategies, however, have contradictory impacts on the ACI values under various levels. For all the ACI-L, ACI-M, and ACI-H, it is the FMOH that has significant influences at almost all its levels. The FMOH and SD are significant for the compliance levels of above 40% and 50%, respectively. The ACI-H can be reduced by 113 days if 80% of people wear facemask. Even, lower FMOH values of 40% and 60% can reduce the ACI-H by 47.0 and 101.2 days, respectively. The intensity of reduction for SD is not as high as that for FMOH. The SD compliance level of 70% brings 83.0 days reduction in ACI-H, which is less than the corresponding value for FMOH of 60%.

FMH use shows statistically significant difference in TI, PoR, and ACI-H; however, the population-level influences are not as strong as for SD and FMOH. For example, using FMH by all people can reduce the infected cases by about 21.6 percent; however, using facemask by only 40% of people out-of-home can reduces the infections by 24.6%. ACI-H is another important attribute which is affected by FMH. Asking people to use mask in-home results in at most 62.4 days reductions in ACI-H which is much lower than those for FMOH.

**Fig 2:**
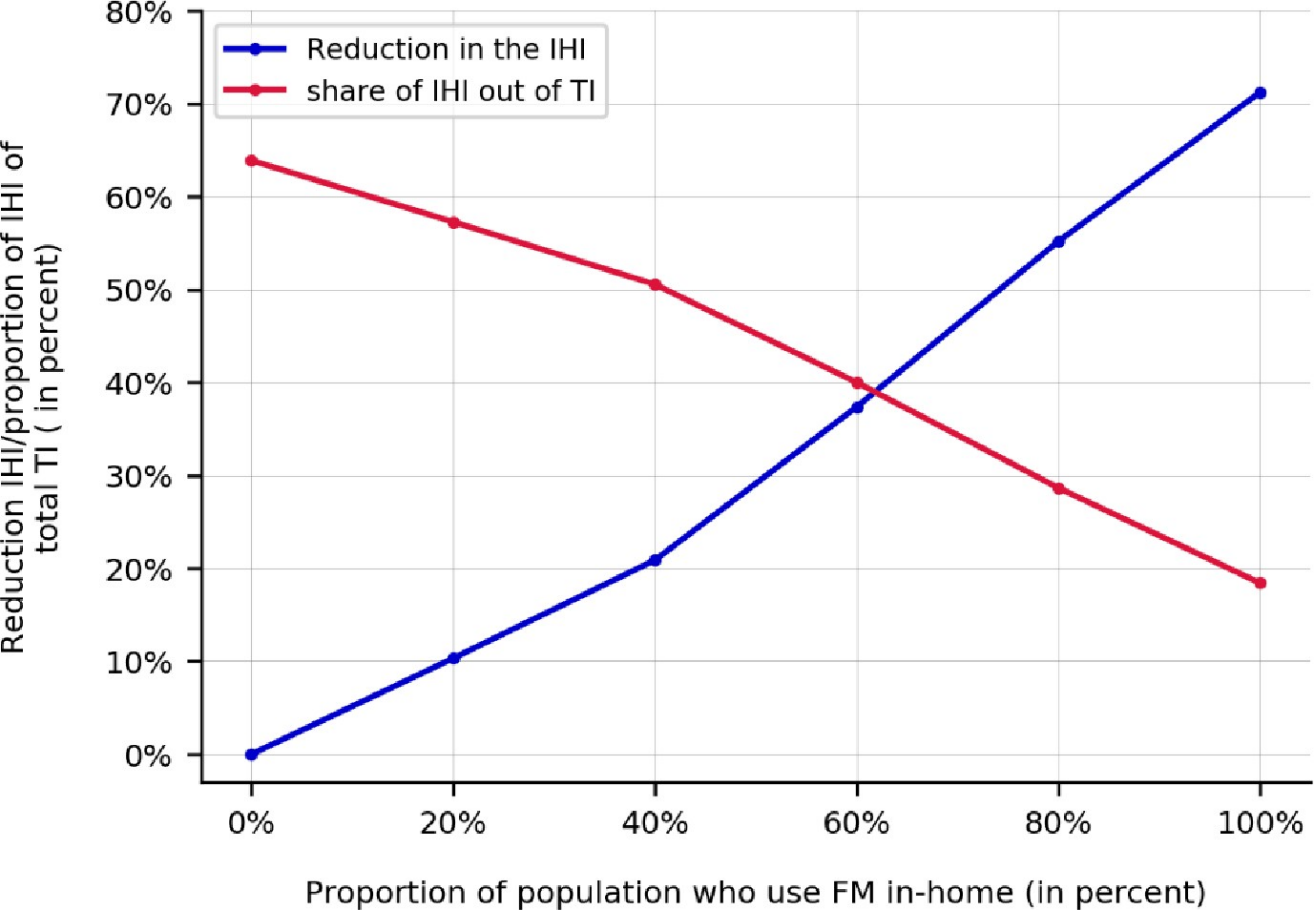
The influence of wearing facemask in-home on the reduction in in-home infections as well as the share of in-home infections over all the infections in the system. Note: while facemask use in-home can reduce the infections in the households by 71.2%, the IHI share is reduced at most by 45.5.

In Fig 2, the influences of FMH on the reduction of in-home infections as well as the share of in-home infections over all infection levels in the system are shown. Contrary to the discussion in S3 File that the FMH can reduce the overall infections across the system (population-level) to the level of 21.6%, where it is found to be effective in reducing the IHI. FMH can reduce the share of in-home infections, on average, by 45.5% percent, from 63.9%, at FMH of 0%, to 18.4%, at FMH of 100%. Although FMH can reduce the IHI share by 45.5%, it reduces the number of in-home infections by 71.2%. To illustrate the figure using an example, if 60% of the population use FM in-home, the number of in-home infections across the population is reduced by 37.4%; however the in-home infections still constitutes 40% of overall infections in the population. Note that the plotted effectiveness values in this figure are the average of the corresponding values across all experiments.

**Fig 3:**
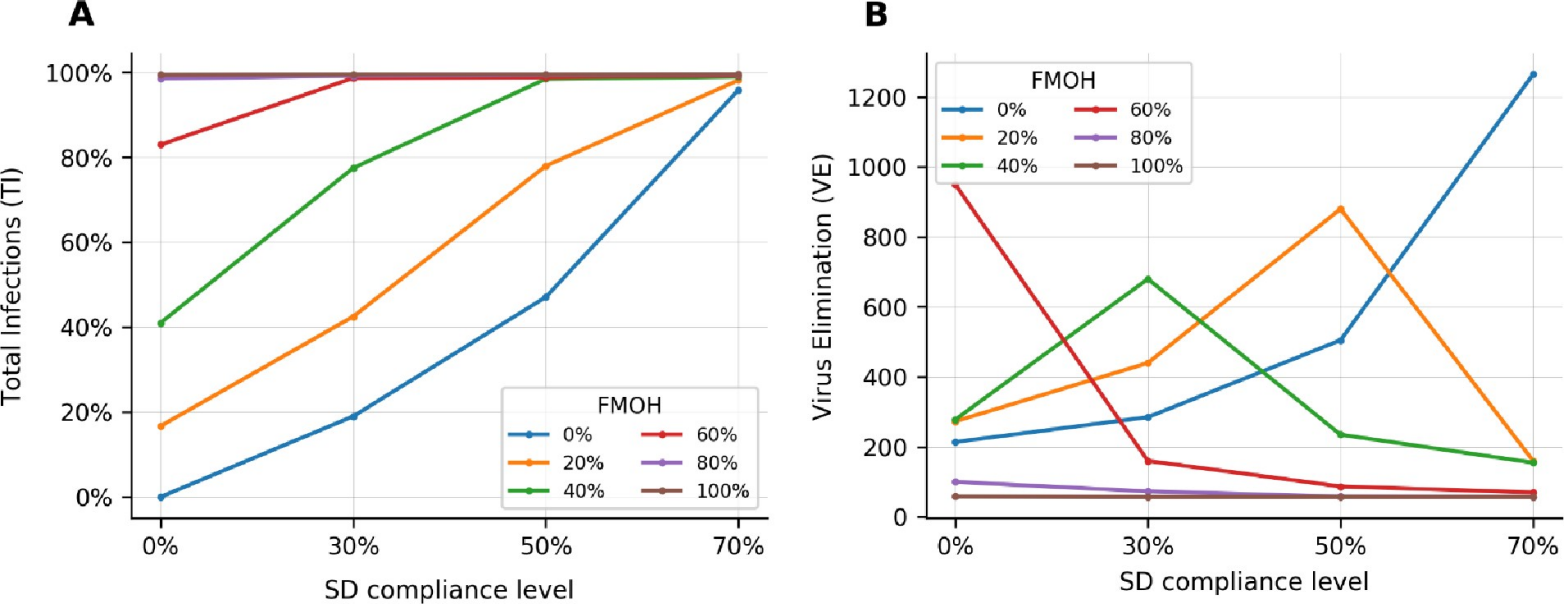
A comparison of different levels of FMOH and their interactions with SD levels when the TL is the same as pre-COVID. (A) The percentage of reduction in the cumulative number of infections (TI), (B) The time it takes the disease spread getting under control (VE). Note: the FMH is 0%.

Fig 3 schematically evaluates the impact of the SD and FMOH control strategies and their interactions in terms of PoR and VE where the estimated marginal means across different starting points are plotted. Fig 3A shows that the SD and FMOH strategies are cumulative in reducing the cumulative number of infections so that low compliance with one can be compensated by higher compliance with the other. At FM rates of 0% and 20%, the epidemic cannot be controlled unless the SD compliance level is 70%. At the SD compliance level of 50%, FM level of 20% does also reduce the number of cases; however, it still puts pressure on the health system. FMOH levels of 60% and higher have the best performance at different levels of SD compliance. The cumulative number of infections shows a significant sensitivity to the lower compliance levels of the SD and FMOH control strategies. The sensitivity reduces by increasing the SD and FMOH levels. The figure also shows that at FMOH over 60% can be a solution for opening the economy. At these levels, the number of infections shows the lowest sensitivity to the SD compliance level.

**Fig 4:**
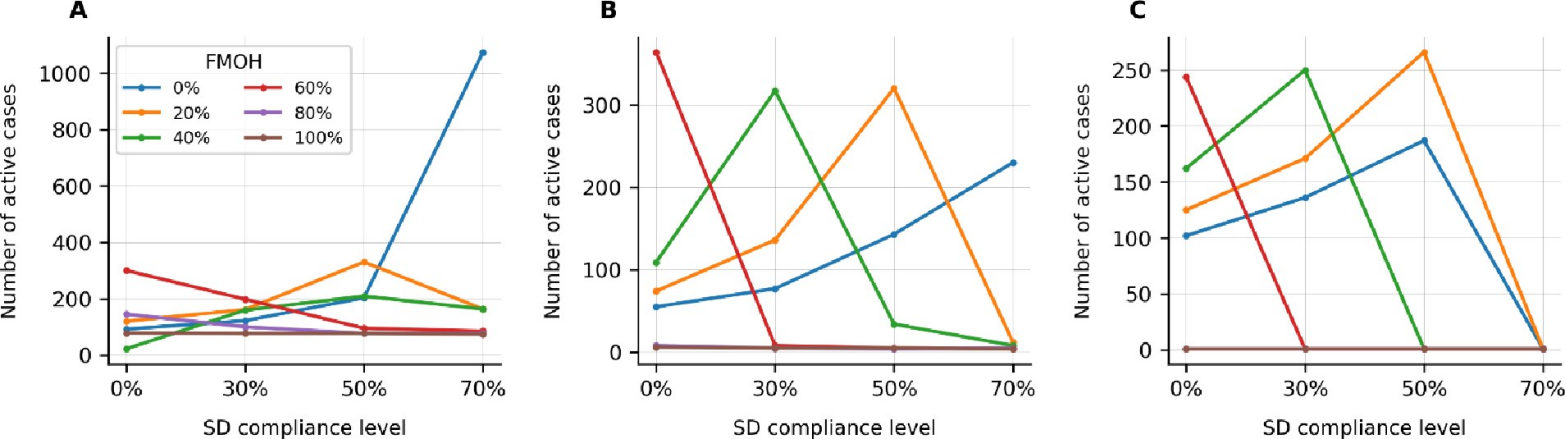
A comparison of the number of active cases across the different levels of FMOH and their interactions with SD levels when the TL is the same as pre-COVID for (A) ACI-L, (B) ACI-M, and (C) ACI-H. Note: the FMH is 0%.

Fig 3B shows the impacts of different combinations of FM and SD compliance levels on the time it takes to control disease transmission. Note that in this figure (and in Fig 4), the absolute values on y-axis should not be the focus of analysis, as they are sensitive to the initial starting points; instead, the sensitivity of the results to the changes in the SD and FMOH levels are the key attributes that we seek for. VE has high interactive effects at the FMOH levels of less than 60% at which the time to eliminate the virus highly depends on the SD compliance level and varies over a wide range. At 0% FMOH levels, the VE has an increasing trend. The VE has a decreasing trend at FMOH levels of 60%, 80% and 100% meaning that the higher SD compliance levels supress the virus earlier. While there is a relatively low number of infections at the FMOH level of 60% and SD compliance level of 0% (concluded from Fig 2A), it takes a long time (about 950 days) to control the disease. The FMOH levels of 20% and 40% show a non-monotonic behaviour. At these levels, the society reaches the herd immunisation and supresses the virus at the low, 0%, and high, 70%, SD compliance levels, respectively. In both cases, the virus is eliminated earlier than the cases where the FMOH level is about 20% or 40% and there is a moderate SD compliance level.

Fig 4 provide a comparison between the number of active cases across the different levels of FMOH and SD. For the FMOH of 80% and higher, irrespective of the SD compliance levels, the active cases are mainly in the ACI-L category which is not still large. On the other side of spectrum, at a FMOH of 40% and lower, the ACI-H is high unless the people comply at high levels of SD; specifically, the minimum SD level of 70% is needed for the FMOH of 20% and lower to remove the pressure on the health system. At the SD compliance levels of 50% and 30%, FMOH of 40% and 60% are needed to obtain the same result.

## 4. Discussion

Simulation, noting that they are too many, of different scenarios on various FM and SD compliance levels informs guidelines for opening-up economies. Other studies have used SEIR modelling, but we modelled individuals using activity-based modelling to analyse the main and interactive effects of the FM, both out-of-home and in-home, and SD control strategies. Under these strategies, the individuals are assumed to be able to participate in daily activities unless they are isolated or quarantined. The relative effectiveness of these strategies is investigated using an ABM where attitudinal, habitual, behavioural, and contextual dynamics of people and systems can be captured to assess different compliance levels of the control strategies.

The results of our analysis support the feasibility of the restrictions relaxation on businesses and other travel restrictions by tightening the rules on the FM and the SD compliance level. FM will result in better disease control than SD, but both together will be even better. Use of FM inside and outside households are both effective. There is no need 100% compliance with either measure to be effective. In countries with high incidence of infection, use of FM and SD together may avoid the need for lockdowns. The FM and SD can reduce the overall infections by up to 54.4% and 24.7%, respectively. This infection reduction potential for SD is fully out-performed with the infection reduction obtainable if only 60% of people wear mask out-of-home. Our analysis revealed that the SD level of 50% is comparable with the FMOH levels of 40%. Also, the SD level of 70% shows comparable results with the FMOH levels of 40% and 60%; while it shows a slightly better performance than FMOH level of 40%, it is outperformed by FMOH levels of above 60%. This finding suggests giving higher priority to facemask usage than SD.

While a high portion of infections happens at home, and high effectiveness of FMH is shown in the literature, only 21.6% of the reductions in the population-level infections is found to be corresponding to the facemask use in-home while the FMOH at a rate of only 40% can result in 24.6% reduction in the PoR. The main reasons explain this crucial finding are:

1) FMOH reduces the disease spread between households (as up-stream) while FMH reduces the spread within household (as down-stream); the influence of inter-household transmissions is higher on the population-level cumulative infections than intra-household transmissions, and

2) if an infected case gets traced and isolated, all her family members get quarantined; this feature makes the influence of FMH less as the quarantining prevents the transmission in the society to a large extent.

Further, owing to the strong effect of both FMH and FMOH on controlling the spread, it is worthwhile to institute comprehensive recommendations for FM use both in-home and out-of-home, depending on the level of community transmission.

The health system is at the forefront of the response to COVID-19. Therefore, the higher the number of days it is under high pressure (ACI-H), or even under medium pressure (ACI-M), is critical. This study indicates that FMOH can considerably reduce the pressure on the health sector; it indicates that a minimum SD level of 70%, FMOH level of 60% is needed if the objective is to remarkably mitigate the pressure on the health system. Although the high SD compliance level of 70% offers better ACI-M and ACI-H than the FMOH of 40%, it is outperformed by the FMOH levels of above 60%. We also found out that SD, at lower compliance levels in particular, is not highly effective. There are also many circumstances in public settings where SD is not possible, predictable or within the control of an individual, as it also depends on the behaviour of others. Wearing facemasks by above 80% of people out-of-home is an alternative solution as it supresses the pandemic irrespective of the SD compliance level.

Universal use of facemasks should be considered as a serious control strategy to enable lifting of restrictions in communities seeking to resume normal activities and could protect people in crowded public settings and within households. Compared to SD, which is negatively correlated with easing restrictions on businesses and activity participations, the FM can efficiently protect people in dense areas. Therefore, combining the SD and FM control strategies can effectively slow down the spread of infection in the community, while restrictions on activity participations are lifted. While SD can reduce the interaction of susceptible and infected persons, a facemask can help minimise the excretion of respiratory droplets from infected individuals who may not even know they are infected. Even if reopening all businesses is not the case, the entire population may want to contribute to reduce transmission; a few months of universal FM would achieve much, albeit with costs. The cost is probably much less, however, than shutting down businesses and schools [24].

We also show that wearing masks by at least 60% of people can be a reasonable public health goal and at the same time a plausible strategy and much easier than enforcing SD. Following SD guidelines is highly correlated with the cultural and behavioural aspects which might not be extensively controllable by local authorities or by individuals. SD is a fuzzy concept which cannot be enforced by only directing people toward acceptable guidelines. In contrast, the FM is a binary concept of yes/no so, controlling and penalising people who avoid using facemasks would be a convenient solution of enforcement to implementation. Also, the use of facemask in places with limited space, such as public transport vehicles, where low compliance for SD guidelines is practiced, can be an effective solution to reduce the infection rates. Participation at these high-risk places can be conditioned on the use of masks. However, facemask shortage is a problem that societies may encounter during the ongoing pandemic. Governments could take responsibility for mass production and distribution of masks to the public rather than solely expecting people prepare and protect themselves.

In fully opening businesses and lifting the restrictions, some societies may find it less likely that they can achieve compliance to the suggested combinations of the recommended SD and FMOH levels to make the virus spread less severe and as a result, control the disease. Therefore, it is still important not to ignore the importance of other risk mitigation strategies, aimed at reducing the number, proximity, and duration of interpersonal contacts, respiratory and hand hygiene measures, and engineering controls such as ventilation in built environments.

The limitations of this study are that system-level performance of FM and SD is influenced by many system parameters (activity-based model here) such as public transit attributes, different network infrastructures, heterogeneous household demographics, and different mobility patterns. Thus, the results in this paper are specific to Sydney greater metropolitan area and may not be the case for other locations. More importantly, it should be highlighted one more time that the relative comparison between the findings can inform the policy not the actual value of each single run of the model under different conditions.

In conclusion, universal FM may assist in pandemic control especially combined with SD. The FM use by at least 60% of people is identified as a feasible strategy for opening the economy, especially owing to the fact that FM compliance is much easier to be enforced than SD. The findings of this research support the potential benefits of a FM strategy along with SD and provide quantitative estimates of their interactive effects on the population-level pandemic attributes to inform effective policy making.

## Supporting information

S1 File Appendix A. (DOCX)

S2 File Appendix B. (DOCX)

S3 File Appendix C. (DOCX)
