# Correction: Facemask and social distancing, pillars of opening up economies

**DOI:** 10.1371/journal.pone.0252633

**Published:** 2021-06-04

**Authors:** The PLOS ONE Staff

## Notice of republication

This article was republished on May 19, 2021, to remove a Supporting Information file that was incorrectly included in the originally published article, and to add the correct Supporting Information files. The article’s Data Availability statement has also been updated to reflect this change. Please download this article again to view the correct version. The publisher apologizes for this error.
