# Supplementary material for: Facemask and social distancing, pillars of opening up economies
Source: PLoS One. 2021 Apr 20;16(4):e0249677. doi: 10.1371/journal.pone.0249677 (PMC8057568; doi:10.1371/journal.pone.0249677)
Supplement: S1 File — (DOCX) [file pone.0249677.s001.docx]

**Appendix A**

The odds ratio (OR) is a measure of association between an exposure and an outcome. The OR represents the odds that an outcome will occur given a particular exposure, compared to the odds of the outcome occurring in the absence of that exposure. The ratio is most commonly used measure in epidemiology and case-control studies [25]. We use an example to show the changes in the OR at different conditions of the system. Consider an extremely simplified situation with 10,000 individuals who are split into two groups, where half wear masks (A) and the other half do not (B). We have one infected individual who goes among A or B. We consider three scenarios with 2,000, 3,000, and 4,000 contacts. Then, we play with per contact efficiency rates.

By estimating the OR values for all these scenarios, the graph below demonstrates the variation of OR values across different parameters of the simulation model. This simulation model is extremely simplified which does not require computer programming and can be conducted in a spreadsheet. Nonetheless, it might be found useful for illustration purposes to show how a large-scale model can be sensitive to different settings and configurations.


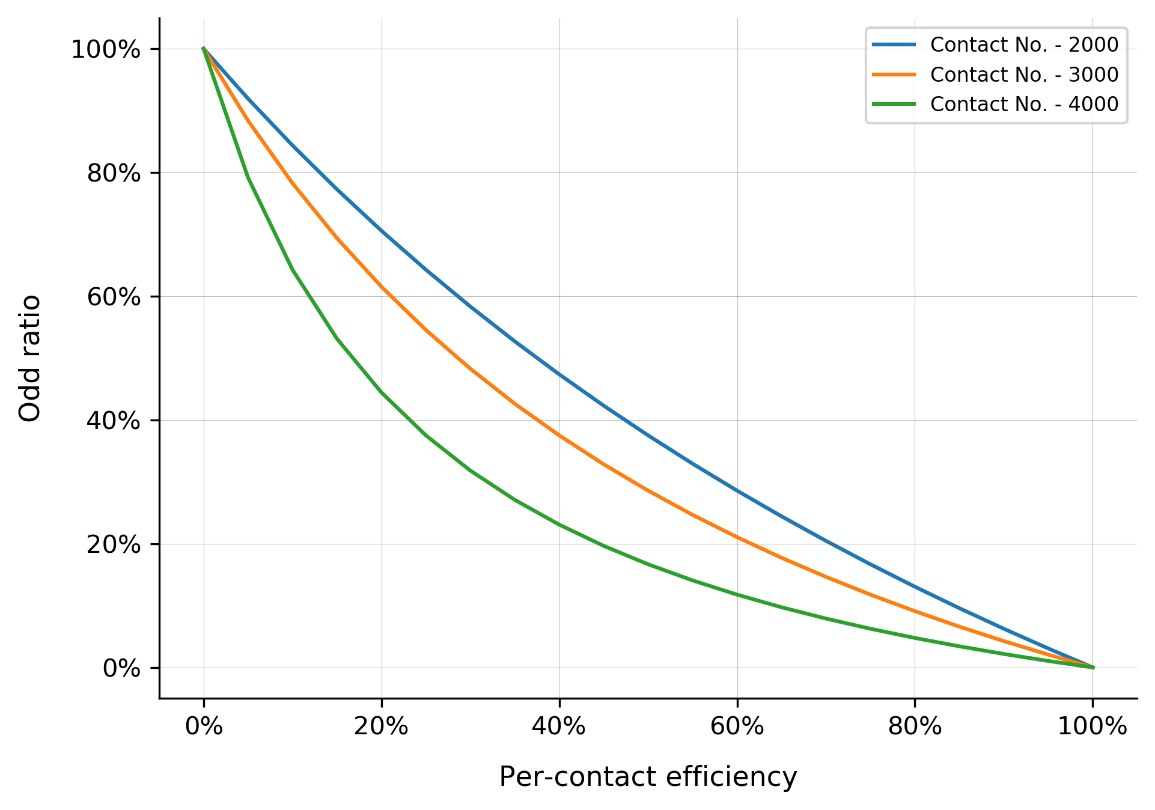


**Figure A1:** Changes of odd ratio across different contact numbers and mask efficiencies

As it can be seen from this diagram, in such a simplified system where everything is tractable, we end up having different efficiencies depending on the configuration, number of contacts, per contact rate, and population. We can also see that we can play with the numbers to provide specific overall efficiency rates if we change the number of contacts or even the per contact rates.
