# Supplementary material for: Facemask and social distancing, pillars of opening up economies
Source: PLoS One. 2021 Apr 20;16(4):e0249677. doi: 10.1371/journal.pone.0249677 (PMC8057568; doi:10.1371/journal.pone.0249677)
Supplement: S2 File — (DOCX) [file pone.0249677.s002.docx]

**Appendix B**

**B.1 Activity-Based Models (ABMs)**

Since 1980, many transport experts and planners have considered ABMs as an alternative to the traditional models in transportation planning. These models contrast the traditional four-stage models in two aspects. First, traditional models typically assume that a traveller’s decisions for making trips (which are the choice of making a trip and the choices of travel destination, mode, and timing of the trip) are independent and the interrelationship between these choices are ignored so trips or tours are modelled. Also, the effect of the relationship between family members on the number of trips and joint trips is not considered. In contrast, ABMs try to consider the chain of trips and model sets of daily activities by considering the relationship between different trips and recognising time and place constraints. The second major distinction is that traditional models use average values of travellers’ characteristics in traffic zones and time periods, which reduces the model's sensitivity to policy changes or investment alternatives. In contrast, ABMs produce a pattern of activities in a disaggregated level for each person. This means activities of each person during the day (and possibly night) are simulated. Afterwards, the place of doing the activity is determined and the chain of trips that establishes the connection between various activities will be created. In this way, all activities will be scheduled in accordance with each other. Also, the activities of each person are determined according to their own socioeconomic characteristics [26]. In summary, although ABMs have obvious advantages over classical models and model the travel behaviours more realistically, these models require more information at a very detailed level. Some of well-known ABM platforms in the literature are Albatross [27], CEMDEP [28], FAMOS [29], GTAModel V4 (TASHA) [7-8,10,30], POLARIS [31-32], MATSim [33], which have been developed based on the basic information provided above.

To solve the lack of detailed information in the development of ABMs, two approaches have been proposed in the literature. First, the incremental implementation, which gradually replaces the components of the existing classical model with the ABM counterparts. It is advantageous for transport organisations because they can invest gradually and evaluate the efficiency of these models. The second approach is transferring and refining implementation, in which a developed ABM for a city is selected and transferred to another city by changing the (private and transit) network, using population distributions, and socio-economic characteristics of the population in the study area. Afterwards, updating various components can be done based on the data availability of the destined city [34]. We have used the transfer and refine implementation approach to develop an ABM for Sydney, Australia. For this purpose, we have transferred the GTA model originally developed for Greater Toronto Area, Canada to Sydney and then refined and calibrated its components and parameters based on the household travel survey for Sydney, the GTFS data, and few macro indicators such as the average number of trips and the temporal distribution of trips along day. This was how the SydneyGMA model has been developed.

SydneyGMA, similar to many other ABMs, is large-scale with many interactive models which are iteratively solved until convergence. The models can be categorised into three main components of population synthesiser, travel demand, and network. The population synthesiser in the SydneyGMA generates the whole population and families. Population synthesiser uses an iterative proportional fitting (IPF) method on the data from Australian Bureau of Statistics. It synthesised individual characteristics (such as age, gender, driving licence, transit pass, employment status, occupation, free parking, student status, employment zone, school zone) and family attributes (such as number of members, number of vehicles, dwelling type) for all 5.8 million Sydney residents.

In SydneyGMA, the travel demand component has various models including a scheduler, and several destination choices and mode choice models. These choice models simulate activities and travel behaviours based on the characteristics of individuals, their families and travel patterns in the city. As the description of each of these models is out of the scope of this appendix, their description has been omitted. The interested readers can refer to [7-12] for further information. Travel times are the determining factors in all the choice models in the demand component. Travel times are estimated using the network component. In the network component, the travel time is determined by assigning the travel demand to different routes in the road network. After determining the travel times over various links of the network, the demand estimation model is iterated, and this process continues until convergence where the travel times of successive iterations reach to the predefined criterion.

In a nutshell, the SydneyGMA simulates the activities of all the people living in Sydney GMA. In addition to the socio-economic characteristics of each person, SydneyGMA determines the details of each person's activities such as purpose, start and end time of each activity, place of activity and travel mode. Owing to such information, i.e. the interaction of people, which is the main factor of disease spread in communities, can be simulated as the tempo-spatial information of all the people is known. Thus, the SydneyGMA model is used in this study to build an activity-based disease spread model to provide rigorous evaluation of various interventions.

**B.2 Activity-based disease spread model**

While SydneyGMA is originally developed to simulate the travel behavior of people, it is extended to model the transmission of the disease in the population while the people participate various activities including work and school and use different modes. We call the extension activity-based disease spread model (ABDSM) which is built on SydneyGMA to simulate the spread of the disease over the social network (see Figure B1). In the ABDSM, a disease spread simulator frequently runs SydneyGMA. Similar to the other ABMs, the SydneyGMA is for daily travel scheduling while the ABDSM should cover the whole lifetime of pandemic which may be few months or years. In the current paper, the ABDSM is used to investigate the effectiveness of various compliance levels of SD and FM use policies in controlling the pandemic.

| *ABDSM*  Disease spread simulator  SydneyGMA  Scheduling timeframe of ABM  Planning horizon of ABDSM |
| --- |
| **Figure B1:** The structure of activity-based disease spread models |

**B.3 Parameters**

Contact rates and infection probabilities are the determinant factors in the spread of disease in SydneyGMA. There are several parameter groups that affect the factors. The parameters can be categorised into 1) travel behaviour-specific parameters, 2) intervention-specific parameters, and 3) disease-specific parameters. The travel behaviour-specific parameters affect out-of-home activity participation rates, destination choices, travel mode choices, the start time, location and duration of out-of-home activity episodes, and contact number for different activity types. This category of parameters is population-, geographic- and demographic-specific; thus, the values of the parameters are different in various ABMs. The travel behaviour-specific parameters are calibrated while developing SydneyGMA; thus, their values are set fixed while calibrating the ABDSM.

The intervention-specific parameters determine the policies that might be applied by policymakers and authorities to slow down the disease spread. These include, but not limited to, the enforcement of business closings, teleworking, and, if applicable, easing the restrictions on businesses; school closures and re-openings; infected case isolation; quarantining of family members; social distancing, facemask use; and the dates when the restrictions are in place. Another intervention-specific parameter is the change of trip generation rates, which is usually ignored in conventional disease spreading models. The intervention-specific parameters can be used for policy analysis to investigate the influence of enforcing various interventions on the transmission of the disease. The parameters are policy sensitive and are used in the paper to determine various compliance levels of SD, FMOH and FMH in simulations. Actually, the sensitivity of the ABDSM to the changes in intervention-specific parameters respond to the research questions of this study.

The disease-specific parameters include incubation period, average time required for an infected individual to recover, and the probabilities of becoming infected (per contacted person), transitioning from infectious to quarantined, infected individuals dying, and transitioning from quarantined to recovered. The parameters are dependent on the disease type and the performance of the health system in Sydney; thus, they should be calibrated against the observed statistics for the transmission of the disease in Sydney GMA. Thus, the disease-specific parameters in SydneyGMA have been at the focus of the ABDSM’s calibration in Najmi et al. [6]; the most important parameters are listed in Table 1. Note that while there are many estimations for the parameters of conventional models such as SIR and SIER in the literature, the parameters are not applicable for the ABDSM in this paper. Also, note that as the ABDSM uses the already calibrated and established SydneyGMA, travel behaviour-specific parameters are assumed correct and usually are not get involved in the calibration of ABDSMs unless the parameters are not originally belonged to the ABMs; the best example is the contact numbers.
