# Supplementary material for: Facemask and social distancing, pillars of opening up economies
Source: PLoS One. 2021 Apr 20;16(4):e0249677. doi: 10.1371/journal.pone.0249677 (PMC8057568; doi:10.1371/journal.pone.0249677)
Supplement: S3 File — (DOCX) [file pone.0249677.s003.docx]

| **Appendix C**  Table C1 The Mean Difference (I-J) between different control strategy levels obtained by two-way MANOVA | | | | | | | | | | | | | | | | | | |  | | | |  |  |  |  |
| --- | --- | --- | --- | --- | --- | --- | --- | --- | --- | --- | --- | --- | --- | --- | --- | --- | --- | --- | --- | --- | --- | --- | --- | --- | --- | --- |
| *Independent variable levels* | |  | Total infections (TI) | | |  | Percentage of reduction (PoR) | | |  | Virus elimination (VE) | | |  |  | Active cases intensity – Low (ACI-L) | | |  | Active cases intensity – Medium (ACI-M) | | |  | Active cases intensity – High (ACI-H) | | |
|  |  |  | Mean Difference^**^  (I-J) | 95% Confidence Interval | |  | Mean Difference^**^  (I-J) | 95% Confidence Interval | |  | Mean Difference^**^  (I-J) | 95% Confidence Interval | |  |  | Mean Difference^**^  (I-J) | 95% Confidence Interval | |  | Mean Difference^**^  (I-J) | 95% Confidence Interval | |  | Mean Difference^**^  (I-J) | 95% Confidence Interval | |
| *Level I* | *Level J* |  |  | Lower Bound | Upper Bound |  |  | Lower Bound | Upper Bound |  |  | Lower Bound | Upper Bound |  |  |  | Lower Bound | Upper Bound |  |  | Lower Bound | Upper Bound |  |  | Lower Bound | Upper Bound |
| ***SD*** | | | | | | | | | | | | | | |  | | | |  | | | |  |  |  |  |
| 0% | 30% |  | 5.41E+05^*^ | 5.06E+05 | 5.76E+05 |  | -12.4^*^ | -13.0 | -11.9 |  | 7.8 | 7.3 | 8.3 |  |  | 1.2 | 1.2 | 1.2 |  | -4.2 | -4.7 | -3.7 |  | 8.8 | 7.8 | 9.8 |
|  | 50% |  | 9.16E+05^*^ | 8.58E+05 | 9.75E+05 |  | -21.0^*^ | -21.7 | -20.3 |  | 33.1 | 32.0 | 34.2 |  |  | -11.1 | -13.0 | -9.2 |  | -1.0 | -1.6 | -0.4 |  | 43.3^*^ | 40.9 | 45.7 |
|  | 70% |  | 1.08E+06^*^ | 1.03E+06 | 1.12E+06 |  | -24.7^*^ | -26.2 | -23.2 |  | 233.9^*^ | 223.0 | 244.8 |  |  | 45.4^*^ | 43.4 | 47.4 |  | 103.9^*^ | 97.5 | 110.3 |  | 83.0^*^ | 78.1 | 87.9 |
| 30% | 0% |  | -5.41E+05^*^ | -5.65E+05 | -5.17E+05 |  | 12.4^*^ | 11.8 | 13.1 |  | -7.8 | -8.2 | -7.4 |  |  | -1.2 | -1.2 | -1.2 |  | 4.2 | 3.8 | 4.6 |  | -8.8 | -9.8 | -7.8 |
|  | 50% |  | 3.75E+05^*^ | 3.56E+05 | 3.94E+05 |  | -8.6^*^ | -9.1 | -8.1 |  | 25.3 | 24.8 | 25.8 |  |  | -12.2 | -14.2 | -10.2 |  | 3.3 | 2.6 | 4.0 |  | 34.5^*^ | 33.4 | 35.6 |
|  | 70% |  | 5.36E+05^*^ | 5.14E+05 | 5.58E+05 |  | -12.3^*^ | -13.1 | -11.5 |  | 226.1^*^ | 217.0 | 235.2 |  |  | 44.2^*^ | 41.8 | 46.6 |  | 108.1^*^ | 101.4 | 114.8 |  | 74.1^*^ | 70.2 | 78.0 |
| 50% | 0% |  | -9.16E+05^*^ | -9.69E+05 | -8.63E+05 |  | 21.0^*^ | 19.9 | 22.1 |  | -33.1 | -34.1 | -32.1 |  |  | 11.1 | 8.2 | 14.0 |  | 1.0 | 0.3 | 1.7 |  | -43.3^*^ | -46.4 | -40.2 |
|  | 30% |  | -3.75E+05^*^ | -3.86E+05 | -3.64E+05 |  | 8.6^*^ | 8.2 | 9.0 |  | -25.3 | -25.8 | -24.8 |  |  | 12.2 | 9.6 | 14.8 |  | -3.3 | -4.7 | -1.9 |  | -34.5^*^ | -36.0 | -33.0 |
|  | 70% |  | 1.61E+05^*^ | 1.56E+05 | 1.66E+05 |  | -3.7^*^ | -3.9 | -3.5 |  | 200.8^*^ | 190.3 | 211.3 |  |  | 56.5^*^ | 50.5 | 62.5 |  | 104.8^*^ | 101.7 | 107.9 |  | 39.6^*^ | 37.7 | 41.5 |
| 70% | 0% |  | -1.08E+06^*^ | -1.12E+06 | -1.03E+06 |  | 24.7^*^ | 23.7 | 25.7 |  | -233.9^*^ | -241.5 | -226.3 |  |  | -45.4^*^ | -48.2 | -42.6 |  | -103.9^*^ | -108.3 | -99.5 |  | -83.0^*^ | -87.8 | -78.2 |
|  | 30% |  | -5.36E+05^*^ | -5.55E+05 | -5.17E+05 |  | 12.3^*^ | 11.7 | 13.0 |  | -226.1^*^ | -235.8 | -216.4 |  |  | -44.2^*^ | -46.2 | -42.2 |  | -108.1^*^ | -113.8 | -102.4 |  | -74.1^*^ | -77.5 | -70.7 |
|  | 50% |  | -1.61E+05^*^ | -1.66E+05 | -1.56E+05 |  | 3.7^*^ | 3.5 | 3.9 |  | -200.8^*^ | -214.3 | -187.3 |  |  | -56.5^*^ | -60.1 | -52.9 |  | -104.8^*^ | -109.1 | -100.5 |  | -39.6^*^ | -40.7 | -38.5 |
| ***FMOH*** | | | | | | | | | | | | | | |  | | | |  | | | |  |  |  |  |
| 0% | 20% |  | 5.62E+05^*^ | 5.28E+05 | 5.97E+05 |  | -12.9^*^ | -13.4 | -12.4 |  | 31.9 | 26.4 | 37.4 |  |  | 15.8 | 12.9 | 18.7 |  | 6.1 | 5.4 | 6.8 |  | 8.5 | 7.3 | 9.7 |
|  | 40% |  | 1.07E+06^*^ | 1.01E+06 | 1.13E+06 |  | -24.6^*^ | -25.6 | -23.6 |  | 119.9^*^ | 108.0 | 131.8 |  |  | 45.6^*^ | 40.0 | 51.2 |  | 27.5^*^ | 26.5 | 28.5 |  | 47.0^*^ | 44.8 | 49.2 |
|  | 60% |  | 1.39E+06^*^ | 1.32E+06 | 1.47E+06 |  | -32.0^*^ | -33.7 | -30.2 |  | 244.7^*^ | 230.5 | 258.9 |  |  | 66.3^*^ | 59.3 | 73.3 |  | 79.1^*^ | 75.0 | 83.2 |  | 101.2^*^ | 98.0 | 104.4 |
|  | 80% |  | 1.42E+06^*^ | 1.35E+06 | 1.50E+06 |  | -32.7^*^ | -33.6 | -31.7 |  | 426.4^*^ | 408.8 | 444.0 |  |  | 152.1^*^ | 145.3 | 158.9 |  | 160.4^*^ | 152.8 | 168.0 |  | 113.0^*^ | 109.4 | 116.6 |
|  | 100% |  | 1.42E+06^*^ | 1.36E+06 | 1.49E+06 |  | -32.8^*^ | -34.1 | -31.5 |  | 448.9^*^ | 432.8 | 465.0 |  |  | 173.6^*^ | 161.6 | 185.6 |  | 160.8^*^ | 153.7 | 167.9 |  | 113.0^*^ | 107.0 | 119.0 |
| 20% | 0% |  | -5.62E+05^*^ | -5.92E+05 | -5.33E+05 |  | 12.9^*^ | 12.2 | 13.5 |  | -31.9 | -36.9 | -26.9 |  |  | -15.8 | -20.6 | -11.0 |  | -6.1 | -6.7 | -5.5 |  | -8.5 | -9.2 | -7.8 |
|  | 40% |  | 5.09E+05^*^ | 4.89E+05 | 5.29E+05 |  | -11.7^*^ | -12.1 | -11.4 |  | 88.0^*^ | 84.0 | 92.0 |  |  | 29.8^*^ | 29.5 | 30.1 |  | 21.5 | 18.9 | 24.1 |  | 38.5^*^ | 37.4 | 39.6 |
|  | 60% |  | 8.30E+05^*^ | 7.91E+05 | 8.69E+05 |  | -19.1^*^ | -19.7 | -18.5 |  | 212.8^*^ | 204.4 | 221.2 |  |  | 50.5^*^ | 49.0 | 52.0 |  | 73.0^*^ | 69.1 | 76.9 |  | 92.7^*^ | 89.5 | 95.9 |
|  | 80% |  | 8.60E+05^*^ | 8.29E+05 | 8.91E+05 |  | -19.8^*^ | -21.1 | -18.5 |  | 394.6^*^ | 379.1 | 410.1 |  |  | 136.3^*^ | 131.6 | 141.0 |  | 154.3^*^ | 147.3 | 161.3 |  | 104.5^*^ | 101.1 | 107.9 |
|  | 100% |  | 8.60E+05^*^ | 8.06E+05 | 9.15E+05 |  | -19.8^*^ | -20.7 | -18.8 |  | 417.0^*^ | 400.6 | 433.4 |  |  | 157.9^*^ | 154.0 | 161.8 |  | 154.7^*^ | 145.1 | 164.3 |  | 104.5^*^ | 98.8 | 110.2 |
| 40% | 0% |  | -1.07E+06^*^ | -1.13E+06 | -1.01E+06 |  | 24.6^*^ | 23.8 | 25.4 |  | -119.9^*^ | -126.5 | -113.3 |  |  | -45.6^*^ | -48.9 | -42.3 |  | -27.5^*^ | -28.6 | -26.4 |  | -47.0^*^ | -49.6 | -44.4 |
|  | 20% |  | -5.09E+05^*^ | -5.38E+05 | -4.79E+05 |  | 11.7^*^ | 11.0 | 12.4 |  | -88.0^*^ | -91.7 | -84.3 |  |  | -29.8^*^ | -30.2 | -29.4 |  | -21.5 | -24.4 | -18.6 |  | -38.5^*^ | -40.1 | -36.9 |
|  | 60% |  | 3.21E+05^*^ | 3.10E+05 | 3.32E+05 |  | -7.4^*^ | -7.8 | -6.9 |  | 124.8^*^ | 118.6 | 131.0 |  |  | 20.7 | 20.1 | 21.3 |  | 51.5^*^ | 48.1 | 54.9 |  | 54.1^*^ | 51.5 | 56.7 |
|  | 80% |  | 3.51E+05^*^ | 3.32E+05 | 3.70E+05 |  | -8.1^*^ | -8.4 | -7.7 |  | 306.6^*^ | 289.8 | 323.4 |  |  | 106.5^*^ | 103.3 | 109.7 |  | 132.8^*^ | 127.0 | 138.6 |  | 65.9^*^ | 62.6 | 69.2 |
|  | 100% |  | 3.51E+05^*^ | 3.31E+05 | 3.72E+05 |  | -8.0^*^ | -8.5 | -7.6 |  | 329.0^*^ | 311.7 | 346.3 |  |  | 128.1^*^ | 121.8 | 134.4 |  | 133.2^*^ | 128.2 | 138.2 |  | 65.9^*^ | 62.6 | 69.2 |
| 60% | 0% |  | -1.39E+06^*^ | -1.46E+06 | -1.32E+06 |  | 32.0^*^ | 30.3 | 33.6 |  | -244.7^*^ | -264.6 | -224.8 |  |  | -66.3^*^ | -70.0 | -62.6 |  | -79.1^*^ | -84.8 | -73.4 |  | -101.2^*^ | -105.7 | -96.7 |
|  | 20% |  | -8.30E+05^*^ | -8.64E+05 | -7.96E+05 |  | 19.1^*^ | 18.2 | 20.0 |  | -212.8^*^ | -220.3 | -205.3 |  |  | -50.5^*^ | -52.2 | -48.8 |  | -73.0^*^ | -79.7 | -66.3 |  | -92.7^*^ | -96.9 | -88.5 |
|  | 40% |  | -3.21E+05^*^ | -3.33E+05 | -3.08E+05 |  | 7.4^*^ | 6.9 | 7.8 |  | -124.8^*^ | -131.1 | -118.5 |  |  | -20.7 | -21.4 | -20.0 |  | -51.5^*^ | -54.9 | -48.1 |  | -54.1^*^ | -55.5 | -52.7 |
|  | 80% |  | 3.05E+04^*^ | 2.92E+04 | 3.19E+04 |  | -0.7^*^ | -0.7 | -0.7 |  | 181.8^*^ | 173.9 | 189.7 |  |  | 85.8^*^ | 83.1 | 88.5 |  | 81.3^*^ | 78.7 | 83.9 |  | 11.8^*^ | 11.4 | 12.2 |
|  | 100% |  | 3.07E+04^*^ | 2.96E+04 | 3.19E+04 |  | -0.7^*^ | -0.7 | -0.7 |  | 204.3^*^ | 199.4 | 209.2 |  |  | 107.4^*^ | 103.3 | 111.5 |  | 81.7^*^ | 79.5 | 83.9 |  | 11.8^*^ | 11.3 | 12.3 |
| 80% | 0% |  | -1.42E+06^*^ | -1.52E+06 | -1.33E+06 |  | 32.7^*^ | 30.7 | 34.6 |  | -426.4^*^ | -453.5 | -399.3 |  |  | -152.1^*^ | -162.3 | -141.9 |  | -160.4^*^ | -166.9 | -153.9 |  | -113.0^*^ | -117.7 | -108.3 |
|  | 20% |  | -8.60E+05^*^ | -9.03E+05 | -8.17E+05 |  | 19.8^*^ | 18.7 | 20.9 |  | -394.6^*^ | -405.9 | -383.3 |  |  | -136.3^*^ | -140.5 | -132.1 |  | -154.3^*^ | -160.1 | -148.5 |  | -104.5^*^ | -108.1 | -100.9 |
|  | 40% |  | -3.51E+05^*^ | -3.65E+05 | -3.38E+05 |  | 8.1^*^ | 7.5 | 8.6 |  | -306.6^*^ | -319.5 | -293.7 |  |  | -106.5^*^ | -109.5 | -103.5 |  | -132.8^*^ | -137.0 | -128.6 |  | -65.9^*^ | -68.6 | -63.2 |
|  | 60% |  | -3.05E+04^*^ | -3.20E+04 | -2.90E+04 |  | 0.7^*^ | 0.7 | 0.7 |  | -181.8^*^ | -190.6 | -173.0 |  |  | -85.8^*^ | -88.7 | -82.9 |  | -81.3^*^ | -82.9 | -79.7 |  | -11.8^*^ | -12.4 | -11.2 |
|  | 100% |  | 2.05E+02 | 1.95E+02 | 2.16E+02 |  | 0.0 | 0.0 | 0.0 |  | 22.5 | 21.6 | 23.4 |  |  | 21.5 | 20.8 | 22.2 |  | 0.4 | 0.4 | 0.4 |  | 0.2 | 0.2 | 0.2 |
| 100% | 0% |  | -1.42E+06^*^ | -1.51E+06 | -1.33E+06 |  | 32.6^*^ | 31.6 | 34.1 |  | -448.9^*^ | -469.7 | -428.1 |  |  | -173.6^*^ | -182.1 | -165.1 |  | -160.8^*^ | -165.3 | -156.3 |  | -113.0^*^ | -119.7 | -106.3 |
|  | 20% |  | -8.60E+05^*^ | -8.86E+05 | -8.35E+05 |  | 19.8^*^ | 19.0 | 20.6 |  | -417.0^*^ | -431.2 | -402.8 |  |  | -157.9^*^ | -164.9 | -150.9 |  | -154.7^*^ | -161.6 | -147.8 |  | -104.5^*^ | -109.7 | -99.3 |
|  | 40% |  | -3.51E+05^*^ | -3.70E+05 | -3.33E+05 |  | 8.0^*^ | 7.7 | 8.4 |  | -329.0^*^ | -346.9 | -311.1 |  |  | -128.1^*^ | -131.6 | -124.6 |  | -133.2^*^ | -137.6 | -128.8 |  | -65.9^*^ | -68.3 | -63.5 |
|  | 60% |  | -3.07E+04^*^ | -3.27E+04 | -2.88E+04 |  | 0.7^*^ | 0.6 | 0.7 |  | -204.3^*^ | -211.7 | -196.9 |  |  | -107.4^*^ | -111.8 | -103.0 |  | -81.7^*^ | -84.2 | -79.2 |  | -11.8^*^ | -12.1 | -11.5 |
|  | 80% |  | -2.05E+02 | -2.18E+02 | -1.93E+02 |  | 0.0 | 0.0 | 0.0 |  | -22.5 | -23.4 | -21.6 |  |  | -21.5 | -22.1 | -20.9 |  | -0.4 | -0.4 | -0.4 |  | -0.2 | -0.2 | -0.2 |
| ***FMH*** |  |  |  |  |  |  |  |  |  |  |  |  |  |  |  |  |  |  |  |  |  |  |  |  |  |  |
| 0% | 20% |  | 2.80E+05^*^ | 2.59E+05 | 3.01E+05 |  | -6.4^*^ | -6.7 | -6.2 |  | 2.4 | 0.4 | 4.4 |  |  | 42^*^ | 41.8 | 42.2 |  | -69.4^*^ | -72.6 | -66.2 |  | 22.6^*^ | 20.4 | 24.8 |
|  | 40% |  | 5.26E+05^*^ | 5.02E+05 | 5.50E+05 |  | -12.1^*^ | -12.6 | -11.6 |  | 44.2^*^ | 40.4 | 48 |  |  | 9.6 | 7.4 | 11.8 |  | -33.2^*^ | -36 | -30.4 |  | 48.8^*^ | 46.8 | 50.8 |
|  | 60% |  | 7.08E+05^*^ | 6.77E+05 | 7.39E+05 |  | -16.3^*^ | -17.3 | -15.2 |  | 138^*^ | 137.4 | 138.6 |  |  | 27.8 | 25.6 | 30 |  | 57.2^*^ | 55.8 | 58.6 |  | 44.8^*^ | 41.8 | 47.8 |
|  | 80% |  | 8.38E+05^*^ | 7.79E+05 | 8.96E+05 |  | -19.2^*^ | -20.7 | -17.8 |  | 167.2^*^ | 162.8 | 171.6 |  |  | 75.8^*^ | 68.6 | 83 |  | 30.6^*^ | 30 | 31.2 |  | 52^*^ | 48 | 56 |
|  | 100% |  | 9.41E+05^*^ | 8.84E+05 | 9.98E+05 |  | -21.6^*^ | -22.4 | -20.8 |  | 81.4^*^ | 79.4 | 83.4 |  |  | 20.6 | 19 | 22.2 |  | -12.2 | -13.2 | -11.2 |  | 62.4^*^ | 57.2 | 67.6 |
| 20% | 0% |  | -2.80E+05^*^ | -2.95E+05 | -2.65E+05 |  | 6.4^*^ | 6.1 | 6.8 |  | -2.4 | -5.2 | 0.4 |  |  | -42^*^ | -42.4 | -41.6 |  | 69.4^*^ | 63 | 75.8 |  | -22.6^*^ | -24.8 | -20.4 |
|  | 40% |  | 2.46E+05^*^ | 2.28E+05 | 2.65E+05 |  | -5.7^*^ | -6.0 | -5.3 |  | 41.8^*^ | 34.8 | 48.8 |  |  | -32.4^*^ | -35 | -29.8 |  | 36.2^*^ | 30.4 | 42 |  | 26^*^ | 25.6 | 26.4 |
|  | 60% |  | 4.28E+05^*^ | 4.01E+05 | 4.55E+05 |  | -9.8^*^ | -10.6 | -9.1 |  | 135.8^*^ | 133.6 | 138 |  |  | -14.2 | -17 | -11.4 |  | 126.6^*^ | 119 | 134.2 |  | 22.2^*^ | 22.2 | 22.2 |
|  | 80% |  | 5.58E+05^*^ | 5.27E+05 | 5.88E+05 |  | -12.8^*^ | -13.3 | -12.3 |  | 165^*^ | 163.4 | 166.6 |  |  | 34^*^ | 29.2 | 38.8 |  | 100^*^ | 95.4 | 104.6 |  | 29.4^*^ | 29 | 29.8 |
|  | 100% |  | 6.61E+05^*^ | 6.26E+05 | 6.97E+05 |  | -15.2^*^ | -16.1 | -14.2 |  | 79.2^*^ | 72.2 | 86.2 |  |  | -21.4 | -23.2 | -19.6 |  | 57.2^*^ | 50.8 | 63.6 |  | 39.8^*^ | 38.8 | 40.8 |
| 40% | 0% |  | -5.26E+05^*^ | -5.56E+05 | -4.96E+05 |  | 12.1^*^ | 11.3 | 12.9 |  | -44.2^*^ | -48 | -40.4 |  |  | -9.6 | -11.8 | -7.4 |  | 33.2^*^ | 30.4 | 36 |  | -48.8^*^ | -50.8 | -46.8 |
|  | 20% |  | -2.46E+05^*^ | -2.62E+05 | -2.31E+05 |  | 5.7^*^ | 5.4 | 5.9 |  | -41.8^*^ | -48.8 | -34.8 |  |  | 32.4^*^ | 29.8 | 35 |  | -36.2^*^ | -42 | -30.4 |  | -26^*^ | -26.4 | -25.6 |
|  | 60% |  | 1.82E+05^*^ | 1.70E+05 | 1.94E+05 |  | -4.2^*^ | -4.4 | -3.9 |  | 94^*^ | 92.6 | 95.4 |  |  | 18.2 | 17.2 | 19.2 |  | 90.4^*^ | 89.2 | 91.6 |  | -4 | -4.2 | -3.8 |
|  | 80% |  | 3.12E+05^*^ | 2.92E+05 | 3.31E+05 |  | -7.1^*^ | -7.5 | -6.8 |  | 123^*^ | 116.6 | 129.4 |  |  | 66.4^*^ | 61.6 | 71.2 |  | 63.8^*^ | 61 | 66.6 |  | 3.4 | 2.8 | 4 |
|  | 100% |  | 4.15E+05^*^ | 3.88E+05 | 4.43E+05 |  | -9.5^*^ | -10.1 | -8.9 |  | 37.4^*^ | 37.4 | 37.4 |  |  | 11 | 11 | 11 |  | 21 | 20 | 22 |  | 13.6^*^ | 11.6 | 15.6 |
| 60% | 0% |  | -7.08E+05^*^ | -7.58E+05 | -6.58E+05 |  | 16.3^*^ | 15.7 | 16.8 |  | -138^*^ | -139 | -137 |  |  | -27.8^*^ | -31.4 | -24.2 |  | -57.2^*^ | -58.6 | -55.8 |  | -44.8^*^ | -46.8 | -42.8 |
|  | 20% |  | -4.28E+05^*^ | -4.44E+05 | -4.12E+05 |  | 9.8^*^ | 9.3 | 10.4 |  | -135.8^*^ | -139 | -132.6 |  |  | 14.2 | 10 | 18.4 |  | -126.6^*^ | -134.4 | -118.8 |  | -22.2^*^ | -22.4 | -22 |
|  | 40% |  | -1.82E+05^*^ | -1.95E+05 | -1.69E+05 |  | 4.2^*^ | 3.9 | 4.4 |  | -94^*^ | -95.4 | -92.6 |  |  | -18.2 | -19.2 | -17.2 |  | -90.4^*^ | -91.6 | -89.2 |  | 4 | 3.8 | 4.2 |
|  | 80% |  | 1.30E+05^*^ | 1.24E+05 | 1.36E+05 |  | -3.0^*^ | -3.1 | -2.8 |  | 29.2 | 24.8 | 33.6 |  |  | 48.2^*^ | 45.6 | 50.8 |  | -26.6^*^ | -29 | -24.2 |  | 7.2 | 6.2 | 8.2 |
|  | 100% |  | 2.33E+05^*^ | 2.19E+05 | 2.47E+05 |  | -5.4^*^ | -5.7 | -5.0 |  | -56.6^*^ | -58.6 | -54.6 |  |  | -7 | -8 | -6 |  | -69.4^*^ | -69.8 | -69 |  | 17.6^*^ | 16 | 19.2 |
| 80% | 0% |  | -8.38E+05^*^ | -8.72E+05 | -8.04E+05 |  | 19.2^*^ | 17.9 | 20.6 |  | -167.2^*^ | -171.4 | -163 |  |  | -75.8^*^ | -83 | -68.6 |  | -30.6^*^ | -31.6 | -29.6 |  | -52^*^ | -55.8 | -48.2 |
|  | 20% |  | -5.58E+05^*^ | -5.95E+05 | -5.21E+05 |  | 12.8^*^ | 12.1 | 13.5 |  | -165^*^ | -166.2 | -163.8 |  |  | -34^*^ | -41 | -27 |  | -100^*^ | -103.6 | -96.4 |  | -29.4^*^ | -30.2 | -28.6 |
|  | 40% |  | -3.12E+05^*^ | -3.34E+05 | -2.89E+05 |  | 7.1^*^ | 6.8 | 7.5 |  | -123^*^ | -129.4 | -116.6 |  |  | -66.4^*^ | -71.2 | -61.6 |  | -63.8^*^ | -66.6 | -61 |  | -3.4 | -4 | -2.8 |
|  | 60% |  | -1.30E+05^*^ | -1.38E+05 | -1.21E+05 |  | 3.0^*^ | 2.8 | 3.1 |  | -29.2 | -33.2 | -25.2 |  |  | -48.2^*^ | -50.8 | -45.6 |  | 26.6^*^ | 24.8 | 28.4 |  | -7.2 | -7.6 | -6.8 |
|  | 100% |  | 1.03E+05^*^ | 9.88E+04 | 1.08E+05 |  | -2.4^*^ | -2.5 | -2.3 |  | -85.8^*^ | -91.4 | -80.2 |  |  | -55.2^*^ | -57.6 | -52.8 |  | -42.8^*^ | -44.2 | -41.4 |  | 10.4 | 9.8 | 11 |
| 100% | 0% |  | -9.41E+05^*^ | -1.01E+06 | -8.72E+05 |  | 21.6^*^ | 20.6 | 22.6 |  | -81.4^*^ | -83.8 | -79 |  |  | -20.6 | -22.4 | -18.8 |  | 12.2 | 11.2 | 13.2 |  | -62.4^*^ | -66.8 | -58 |
|  | 20% |  | -6.61E+05^*^ | -6.87E+05 | -6.35E+05 |  | 15.2^*^ | 14.4 | 16.0 |  | -79.2^*^ | -85.8 | -72.6 |  |  | 21.4 | 19.2 | 23.6 |  | -57.2^*^ | -61.6 | -52.8 |  | -39.8^*^ | -41.6 | -38 |
|  | 40% |  | -4.15E+05^*^ | -4.38E+05 | -3.92E+05 |  | 9.5^*^ | 9.1 | 9.9 |  | -37.4^*^ | -37.4 | -37.4 |  |  | -11 | -11 | -11 |  | -21 | -22 | -20 |  | -13.6^*^ | -15.6 | -11.6 |
|  | 60% |  | -2.33E+05^*^ | -2.48E+05 | -2.18E+05 |  | 5.4^*^ | 5.1 | 5.6 |  | 56.6^*^ | 54.2 | 59 |  |  | 7 | 6.2 | 7.8 |  | 69.4^*^ | 69.2 | 69.6 |  | -17.6^*^ | -18.8 | -16.4 |
|  | 80% |  | -1.03E+05^*^ | -1.10E+05 | -9.73E+04 |  | 2.4^*^ | 2.3 | 2.5 |  | 85.8^*^ | 81.6 | 90 |  |  | 55.2^*^ | 52.2 | 58.2 |  | 42.8^*^ | 40.2 | 45.4 |  | -10.4 | -11.4 | -9.4 |
| * The mean difference is significant at the .05 level.  ** The table provides the mean difference of the level J’s response variable from the level I’s response value. | | | | | | | | | | | | | | | | | | | | | | | | | |  |
